# Heel raises versus prefabricated orthoses in the treatment of posterior heel pain associated with calcaneal apophysitis (Sever's disease): study protocol for a randomised controlled trial

**DOI:** 10.1186/1757-1146-4-S1-P28

**Published:** 2011-05-20

**Authors:** Alicia M James, Cylie M Williams, Terry P Haines

**Affiliations:** 1 Cardinia Casey Community Health Service, Southern Health, Cranbourne, Australia; 2 Peninsula Community Health Service - Frankston, Peninsula Health, Frankston, Australia; 3 Allied Health Clinical Research Unit, Southern Health, Cheltenham, Australia

## Background

Posterior Heel pain can present in children of 8 to 14 years, associated with or clinically diagnosed as Sever's disease, or calcaneal apophysitis. Presently, there are no comparative randomised studies evaluating treatment options for posterior heel pain in children with the clinical diagnosis of calcaneal apophysitis or Sever's disease. This study seeks to compare the clinical efficacy of some currently employed treatment options for the relief of disability and pain associated with posterior heel pain in children.

## Method

*Design*: Factorial 2 × 2 randomised controlled trial with monthly follow-up for 3 months. *Participants:* Children with clinically diagnosed posterior heel pain possibly associated with calcaneal apophysitis/Sever's disease (n = 124). *Interventions:* Treatment factor 1 will be two types of shoe orthoses: a heel raise or prefabricated orthoses. Both of these interventions are widely available, mutually exclusive treatment approaches that are relatively low in cost. Treatment factor 2 will be a footwear prescription/replacement intervention involving a shoe with a firm heel counter, dual density EVA midsole and rear foot control. The alternate condition in this factor is no footwear prescription/replacement, with the participant wearing their current footwear. *Assessment:* Foot Posture Index and Lunge Test (Inclinometer testing). *Outcomes:* Oxford Foot and Ankle Questionnaire and the Faces pain scale.

## Discussion

This will be a randomised trial to compare the efficacy of various treatment options for posterior heel pain in children that may be associated with calcaneal apophysitis also known as Sever's disease.

